# Computing power of quantitative trait locus association mapping for haploid loci

**DOI:** 10.1186/1471-2105-10-261

**Published:** 2009-08-23

**Authors:** Derek Gordon, Andrew R Zinn

**Affiliations:** 1 Department of Genetics, Rutgers University, 145 Bevier Road, Piscataway, NJ 08854, USA; 2 McDermott Center for Human Growth and Development, University of Texas Southwestern Medical School, 5323 Harry Hines Blvd, Dallas, TX 75390-8591, USA

## Abstract

**Background:**

Statistical power calculations are a critical part of any study design for gene mapping. Most calculations assume that the locus of interest is biallelic. However, there are common situations in human genetics such as X-linked loci in males where the locus is haploid. The purpose of this work is to mathematically derive the biometric model for haploid loci, and to compute power for QTL mapping when the loci are haploid.

**Results:**

We have derived the biometric model for power calculations for haploid loci and have developed software to perform these calculations. We have verified our calculations with independent mathematical methods.

**Conclusion:**

Our results fill a need in power calculations for QTL mapping studies. Furthermore, failure to appropriately model haploid loci may cause underestimation of power.

## Background

Statistical power calculations are a critical part of any study design for gene mapping. With regards to quantitative trait locus (QTL) mapping, it is typically assumed that there are two allele at the QTL, and hence three genotypes (e.g., see Lynch and Walsh [1]). For each genotype, there is a corresponding mean quantitative value that is determined by the biometric model developed by Falconer and others [2]. To determine the mean of the heterozygote, we need a parameter known as the dominance parameter. Software to compute power for the biallelic situation has been made available in the Genetic Power Calculator, developed by Purcell et al. [3] However, for haploid organisms or loci, there is no such parameter because there are no heterozygotes. Thus, there is a different set of calculations necessary to compute power for association. There has been an extensive amount of literature written on the analysis of haploid data (see, e.g., Wu et al. [4,5], Tulsieram et al. [6]) To our knowledge, there has been no power calculator developed for QTL mapping with haploid loci. This is an oversight, since there are several common situations in humans involving loci that are haploid or hemizygous [7,8].

The purpose of this work is to mathematically derive the biometric model for haploid loci, and to compute power for QTL mapping when the loci are haploid.

## Implementation

The program for computing power is written in C++ and may be created on any computer that has a C++ compiler, for example those computers with a Unix or Linux operating system.

**Figure 1:**
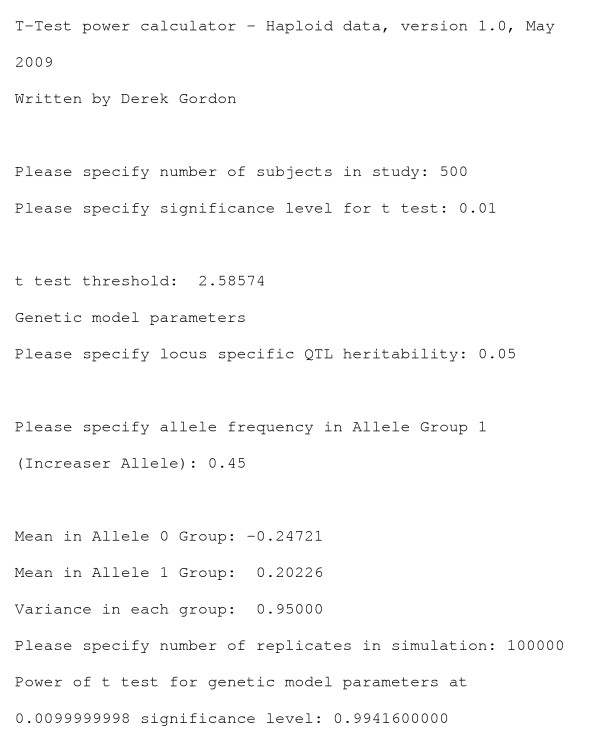
**Example run with program T-Test power calculator – Haploid data**.

After making the executable, the user types the phrase "power-ttesthap" to implement the program. The user is queried for the sample size of the study. Next, the user is asked for the significance level at which simulation power is to be computed. When this value is entered, the threshold for the *t*-test is computed and reported to the screen. Following this, the user is asked about the locus-specific quantitative trait heritability *Q*. When this value is entered, the program determines the means of the allele 0 group and the allele 1 group, as well as the variance in each group. Finally, the user is asked to specify the number of replicates for which power is computed. We recommend at least 100,000 replicates. See Figure 1 for an example of how data are entered, and see additional file 1. Below, we place an example run.

## Results and discussion

### Calculations of simulation power as function of Locus specific QTL heritability and increaser allele frequency

**Figure 2:**
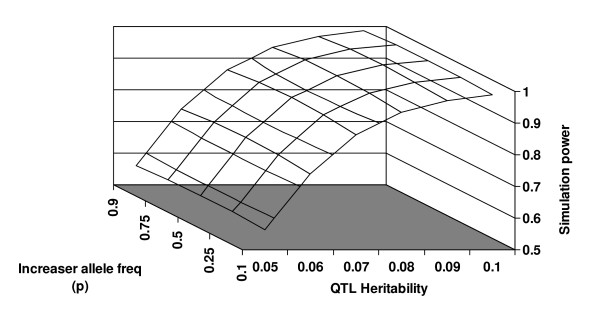
**Simulation power results**. In this figure, we present simulation power results as a function of the increaser allele *p *and the Locus specific QTL heritability *Q*. The total sample size is 500 subjects and the significance level is 1 × 10^-6^.

In Table 1, we provide an abbreviation of all the variables used in the Results section. In Figure 2, we plot the simulation power as a function of the Locus specific QTL heritability and the increaser allele frequency, using the ranges of *Q *and *p *that we specified in the Methods. There are several conclusions we may draw from studying this graph. The first is that, as *Q *increases, the power increases for any value of *p*. The second conclusion is that, if *Q *is held constant, the power differs minimally over the range of *p*. In fact, if we consider the set of consecutive differences ((power for *Q *= 0.05 and *p *= 0.25) – (power for *Q *= 0.05 and *p *= 0.1)), ((power for *Q *= 0.05 and *p *= 0.5) – (power for *Q *= 0.05 and *p *= 0.25)),..., ((power for *Q *= 0.1 and *p *= 0.9) – (power for *Q *= 0.1 and *p *= 0.75)), the maximum difference is 0.012, and the average difference is -0.00025. The maximum difference occurs for the difference ((power for *Q *= 0.07 and *p *= 0.25) – (power for *Q *= 0.07 and *p *= 0.1)). We note that the power was calculated using the executable file that is compiled from the material in Additional File 2.

**Table 1:** List of Abbreviations

*Variable*	**Description**
*N*	Sample size
*Á*	Significance level
*T*	Threshold for *t *test (uses *N *and *α*)
*Q*	Variance of quantitative trait locus (QTL)
*P*	Frequency of QTL increaser allele
*μ*_0_	Mean QTL value in group "0" (those with non-increaser allele)
*μ*_1_	Mean QTL value in group "1" (those with increaser allele)
*R*	Number of replicates

By contrast, if we consider the set of consecutive differences ((power for *Q *= 0.06 and *p *= 0.1) – (power for *Q *= 0.05 and *p *= 0.1)), ((power for *Q *= 0.07 and *p *= 0.1) – (power for *Q *= 0.06 and *p *= 0.1)),..., ((power for *Q *= 0.1 and *p *= 0.9) – (power for *Q *= 0.09 and *p *= 0.9)), the maximum difference is 0.182 and the average difference is 0.085. The maximum difference occurs for ((power for *Q *= 0.06 and *p *= 0.75) – (power for *Q *= 0.05 and *p *= 0.75)).

Another result from this graph is that power of at least 80% may be achieved when the Locus specific QTL heritability *Q *is at least 0.07, suggesting that genes for QTLs with reasonable variance may be mapped even when the significance level is stringent.

### Real data example – EFHC2 gene SNP rs7055196 typed in 45, X Turner Syndrome subjects

Zinn et al. genotyped 97 45, X Turner Syndrome subjects at the SNP rs7055196. They also phenotyped the subjects for Facial Affect Fear Recognition score and performed a *t*-test to determine whether there was a significant difference in the scores based on the allele at the rs7055196 locus [9]. They found no significant difference among the two groups (49.8 +/- 26.5 for those with A allele versus 46.2 +/-26.1 for those with G allele; p-value = 0.67; see Figure 1 of Zinn et al.).

**Figure 3:**
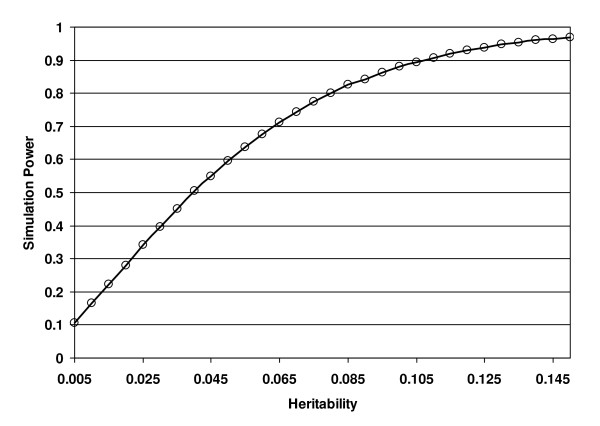
**Simulation power results as a function of locus-specific QTL heritability for EFHC2 gene SNP rs7055196 example**. In this figure, we present simulation power results as a function of the Locus specific QTL heritability *Q*. The total sample size is 97 subjects, the increaser allele frequency is 0.87 and the significance level is 0.05.

Because there are 11 subjects with the G allele and 86 subjects with the A allele, we estimate the increaser allele frequency to be 0.87. The sample size we consider is 97. We specify a range of locus-specific QTL heritabilities from 0.005 to 0.15 in increments of 0.005. Since we are only performing a single test, we consider a significance level of 0.05. We present our results in Figure 3, where each simulation data point is created using 100,000 replicates. In that figure, we see that power increases as the heritability goes up (similar to Figure 2). Also, a heritability of 0.04 gives a simulation power of approximately 0.50. These results suggest that the heritability is lower rather than higher.

## Conclusion

To our knowledge, this is the first work to determine the power of QTL mapping for haploid loci or organisms. We compute power by simulation, and our results with exact analytic power suggest that the power by simulation is highly accurate. The most common application of these results is SNP association studies of loci on the X or Y chromosomes in male subjects. Other situations where haploidy arises in humans include chromosome disorders such as 45,X Turner syndrome [7] and segmental aneuploidies such as Cri-du-Chat (5p-) syndrome [8]. Last, the power to map QTLs in haplo/diploid organisms such as *Saccharomyces cerevisiae *can be increased by studying organisms in the haploid state [10].

Natural extensions of this work are situations such as gene-gene interactions (epistasis of different kinds), gene-environment interactions, and multi-trait mapping. This is the subject of future research. The purpose of this work is to provide researchers with the simplest non-trivial example of power calculations for haploid data.

## Availability and requirements

**Project name**: *t*-test power calculator for haploid data

**Project home page**: 

**Operating system(s)**: Unix Solaris, Linux

**Programming language**: C++

**Other requirements**: None.

**License**: None.

**Any restrictions to use by non-academics**: None.

## Authors' contributions

DG and ARZ conceived of the study and wrote the manuscript. DG created the software program. Both authors read and approved the final manuscript.

## Supplementary Material

Additional file 1 **Methods for haploid power**. This file provides the description of the method used for calculating power. Click here for file

Additional file 2 **Files for compiling power-ttesthap (executable)**. This file containing the information needed to produce the executable that computes haploid power for a fixed sample size. Click here for file
