# Supplementary material for: Computing power of quantitative trait locus association mapping for haploid loci
Source: BMC Bioinformatics. 2009 Aug 23;10:261. doi: 10.1186/1471-2105-10-261 (PMC2738682; doi:10.1186/1471-2105-10-261)
Supplement: Additional file 1 — Methods for haploid power. This file provides the description of the method used for calculating power. [file 1471-2105-10-261-S1.doc]

**Methods**

*Simulation power*

To compute simulation power, we must specify the test statistic we use, the sample size, the significance level, the proportion of individuals with the larger mean quantitative trait locus (QTL) value, and the number of replicates [1]. All notation in this section may be found in the List of Abbreviations. The test statistic we consider is the *t* test for differences of means. With the sample size and significance level given, we can determine the corresponding *t* test threshold *T*. In our application, we will test whether the mean QTL values for the subjects with one allele at a SNP locus differ from the mean QTL values for the sample of subjects with the other allele at the locus. For example, the QTL could underlie cognitive trait values for Turner syndrome subjects [2, 3].

To determine the power, we must specify the means in each group and the variance. To do this, we use the decomposition of genetic variance model first proposed by Fisher [4]. While Fisher considered three genotypes at a locus, we need only consider the simpler situation of two alleles at a SNP locus, since this work focuses on loci that are haploid. We assume, without loss of generality that the QTL has a standard normal distribution with mean 0 and variance 1. Let $Q$ represent the locus specific QTL heritability, that is, the proportion of the total variance in the population due to the SNP being studied. Let *p* be the frequency of the allele that increases the phenotypic measurement (e.g., higher cognitive value). Using the biometric model described by Falconer and others [5], it is straightforward to show that the mean $\mu_{1}$ of the subjects with the “increaser” allele is $m+a$, where and $m=pa+(1-p)(-a)=a(2p-1)$. Similarly, the mean $\mu_{0}$ of the subjects with the other allele is$m-a$. Finally, the variance of each group is the residual variance,$1-Q$. Once we specify $Q$ and *p*, we can compute$\mu_{0},\mu_{1}$, and the common variance $1-Q$.

Each replicate has a randomly determined number of individuals with the non-increaser allele and the increaser allele. The random number is determined using a Bernoulli random number generator where the probability of success is $\mu_{1}$and the total sample size is *N.* In the second stage of randomization, each individual is assigned a quantitative trait value based on its group’s mean and variance, where the random number generator is a normal generator with mean $\mu_{i},i=0\text{ or 1}$, and variance 1- *Q.* After each individual in a replicate is assigned an allele and a quantitative number, a *t* test is performed on the two groups. The total proportion of replicates (out of *R*) with a *t* test exceeding the value *T* is the *simulation power*. The reason for doing simulations is that the gold standard answer of analytic power becomes difficult to compute as the sample size increases.

*Calculations of simulation power as function of Locus specific QTL heritability and increaser allele frequency*

We specify a sample size *N* of 500 and a locus-wise significance level of $1\times10^{-6}$. This significance level is consistent with current and future SNP genotyping platforms. We consider a range of increaser allele frequencies *p*: 0.1, 0.25, 0.5, 0.75 and 0.9. We also consider a range of *Q* values: 0.05, 0.06, 0.07, 0.08, 0.09, and 0.10. In each simulation, the number of replicates is 100,000.

*Real data example – EFHC2 gene SNP rs7055196 typed in 45, X Turner Syndrome subjects*

In this example, 97 45, X Turner Syndrome subjects are genotyped for the SNP rs7055196 in the gene EFHC2 [3], which is a candidate gene for the quantitative trait fear recognition [2, 6]. We consider a range of locus-specific QTL heritabilities that appear consistent with the findings of the *t*-test performed by Zinn et al. [3]

1. Cohen J: **Statistical power analysis for the behavioral sciences**, Second edn. Hillsdale, New Jersey: Lawrence Erlbaum Associates; 1988.

2. Weiss LA, Purcell S, Waggoner S, Lawrence K, Spektor D, Daly MJ, Sklar P, Skuse D: **Identification of EFHC2 as a quantitative trait locus for fear recognition in Turner syndrome**. *Hum Mol Genet* 2007, **16**(1):107-113.

3. Zinn AR, Kushner H, Ross JL: **EFHC2 SNP rs7055196 is not associated with fear recognition in 45,X Turner syndrome**. *Am J Med Genet B Neuropsychiatr Genet* 2008, **147B**(4):507-509.

4. Fisher RA: **The correlation between relatives on the supposition of Mendelian inheritance**. *Trans Royal Soc Edinburgh* 1918, **52**:399-433.

5. Falconer DS, Mackay TFC: **Introduction to Quantitative Genetics**, Fourth edn. San Francisco: Benjamin Cummings; 1996.

6. Good CD, Lawrence K, Thomas NS, Price CJ, Ashburner J, Friston KJ, Frackowiak RS, Oreland L, Skuse DH: **Dosage-sensitive X-linked locus influences the development of amygdala and orbitofrontal cortex, and fear recognition in humans**. *Brain* 2003, **126**(Pt 11):2431-2446.
